# The impact of psychological capital on green and organizational performance: Empirical research measuring psychological and subjective health of green building industries

**DOI:** 10.3389/fpubh.2022.1030028

**Published:** 2023-01-19

**Authors:** Si-Xu Chen, Yuan Tang

**Affiliations:** ^1^College of Engineering, City University of Hong Kong, Kowloon, Hong Kong SAR, China; ^2^School of Management, Sichuan University of Science and Engineering, Zigong, China

**Keywords:** green building (GB), psychological capital (PC), psychological health (PH), green innovation ambidexterity (GIA), subjective health (SH), green performance (GP), organizational performance (OP), self-determination theory (SDT)

## Abstract

**Introduction:**

Green building (GB) technologies have been introduced to reduce the negative effect of the building and construction industry on the economy, the ecosystem, and society. A more thorough and accurate knowledge of the factors for implementation is required to stimulate the wider adoption of GB technologies. The theoretical framework for this study is developed using psychological capital (PC), self-determination theory (SDT), and green theory. The study addresses gaps in the research in this field. The effect of PC on psychological health (PH), green innovation ambidexterity (GIA), and subjective health (SH), and the impact of GIA on green performance (GP) are determined. The effect of PH and SH on organizational performance (OP) is also determined.

**Methods:**

The information for this study is collected from a variety of sources in the Chinese GB industry. The subjects for this study are Chinese employees of GB-based companies. Using a convenience sampling method, a total of 504 employees were selected. The employees' answers to a restricted, self-administered survey are used to generate the data.

**Results:**

Data from this study shows that PC has a significant effect on GIA, SH, and PH and that GIA has a substantial impact on GP. Both SH and PH significantly affect OP.

**Discussion:**

This study encourages managers in the green building industry to support fresh concepts in order to gain a competitive edge by implementing and promoting innovative cultures, especially in terms of service delivery and customer understanding, because innovation plays a critical role in these industries.

## 1. Introduction

Green building (GB) technologies have been introduced to reduce the adverse effects of the building and construction industry on the economy, the ecosystem, and society. To stimulate the wider adoption of GB technologies, a more thorough and accurate knowledge of the factors for implementation is required. These negative effects are mitigated by the use of GB technologies, and the construction sector has increased the sustainable development of its operations through the use of numerous GB technologies (1). Taiwan's GB certification system comprising Energy conservation, Environment, Waste Control and Health (EEWH) has been implemented since 1999 (2). Furthermore, Taiwan has also listed GB industries as one of the four emerging industries in 2010. Taiwan aims to promote the GB industry to enhance architectural technologies and lessen carbon discharges (3). To “meet the requirements of the present without jeopardizing the potential of subsequent generations to fulfill the necessities,” sustainable development is important (4). GB is defined by Ahmad et al. (5) as technology solutions that are implemented in a building layout to produce a sustainable final product, which includes green roof technology, solar power systems, and envelope thermal efficiency. GB increases the ecological, societal, and economic efficiency of buildings. These are three critical factors in addressing the requirement for sustainability in the construction sector (4). GB involves developing structures and employing methods that are sustainable and resource-efficient across the life cycle of a building, in terms of site selection to the concept, building, administration, maintenance, and restoration (6). During the design and construction process, GB uses recyclable materials, less energy, less water, and resource-efficient methods that incorporate water-sensitive design and reduce the vulnerability to flooding. Incorporating GB also reduces the emission of pollutants to water, soil, and air and reduces light and noise pollution, so negative environmental impacts are significantly reduced (7). This sustainable building method also has economic and social implications. Sociologically, GB enhances the conditions for the lives and work of citizens. Economically, GB provides owners or occupants with lifetime cost benefits. Buildings that use a GB design are rented or purchased at a greater rate, so earnings are increased (8). GB has ecological, financial, and sociological benefits (9).

Green innovation ambidexterity (GIA) is essential for sustainability in the context of increasing contamination of the environment and more demanding environmental restrictions (10). GIA significantly increases the ecological, economic, and societal benefits for a business and addresses environmental challenges (10, 11). Only some businesses have the necessary resources and data to embrace GIA, so a significant number of enterprises look beyond their organizations for GIA knowledge and tools (12). This study categorizes GIA as either green exploratory innovation (GEP) or green exploitative innovation (GET) (13). GEP involves the pursuit of a new understanding of green behaviors and the development of innovative products and technologies (14, 15). GET involves upgrading existing green knowledge and green technology to improve current services and products (14). The development and implementation of innovation depend on a company's capital resources (16). Human resources, psychological capital (PC), and social capital have replaced conventional financial investment (17). PC stimulates innovation within enterprises by establishing a favorable setting and is the subject of numerous studies (17–19). Luthans et al. (20) noted that PC includes self-efficacy (SE), hope (HO), optimism (OPT), and resilience (RE), and it enables individuals to be more imaginative and creative in their tasks so assessing an individual's psychological capital is a crucial strategy for fostering innovation (17). Few studies have determined any link between PC (self-efficacy, trust, optimism, and resiliency) and GIA. This study determines the impact of psychological capital on GIA within the environment of the green construction industry (16). There is an increasing trend in the service sector toward studying other elements that may impact green performance (GP). Asadi et al. (21) studied the significance of green innovation in GP in the Malaysian service industry. In terms of the service business, GIA is defined as procedures, commodities, concepts, and management techniques that produce competitive benefits while minimizing any environmental harm that is caused by service industry enterprises (22). Therefore, this study also determines the impact of GIA and GP.

Using self-determination theory (SDT) (23) and its applications to the professional workplace, this study builds on research and the knowledge base for positive organizational behavior. SDT's fundamental premise is that all humans have an inherent propensity to be creative and to develop an expanded and solidified sense of identity (24). They wish to establish relationships with themselves, others, and groups in their surroundings. Individual wellbeing and interpersonal growth are strengthened when the needs for independence, competency, and belongingness are met. Therefore, this study considers PC as a determinant of subjective health (SH) (25). Hence, the effect of PC on the SH of individuals is determined in this study. Furthermore, psychological health (PH) is an aspect of the discipline of positive psychology and involves the cultivation of positive emotions that optimize the functionality and experiences of persons (26). During the past decade, a great deal of study on PH has examined the concept map for this topic. Ryan and Deci (26) proposed two major categories of study: those concerning satisfaction (hedonic wellbeing) and those concerning individual capability (eudaimonic wellbeing) (27). This study also measures the impact of PC on the PH of employees.

There is no certainty that increased SH for employees results in increased work performance or organizational profitability. Within workspaces, team dynamics play an important role, so negative emotions from one worker can spread to others and affect the team's efficiency. Employers' attempts to boost SH involve expenditure, so higher productivity might not have a net positive effect on the company's financial standing. Individual studies determine the relationships between organizational performance and wellbeing. There is limited empirical research on the connection between individuals' organizational performance (OP) and SH at the workplace or company level. No studies verify this relationship using a nationally representative sample for the service and manufacturing industries. This study contributes to the body of knowledge in this field by determining whether there is a strong link between variations in an individual's SH—aggregating to the organizational level—and variations in OP, using a nationally representative sample that permits generalization (28). Studies have shown that HRM methods generally result in beneficial organizational results (29). Still, academics have demonstrated HR policies' contending and often contradictory nature in promoting PH and OP (30). Therefore, this study determines the impact of SH and PH on OP in the context of the green building industry.

In terms of the practical implications of this study, managers in the green building industry are encouraged to support fresh concepts in order to gain a competitive edge by implementing and promoting innovative cultures, especially in terms of service delivery and customer understanding, because innovation plays a critical role in these industries. In terms of the intra-correlations between the PC dimensions, managers can improve each component by fostering and enforcing a productive work environment in the workplace to improve PH, SH, and GIA. This study also contributes to the theory by using green theory, self-determination theory, and PC to develop a theoretical framework. It also addresses research gaps in that it measures the impact of PC on GIA, SH, and PH and it measures the impact of GIA on GP. It also determines the effect of SH and PH on OP.

## 2. Theoretical background

### 2.1. Psychological capital

PC is an element of organizational behavior (31). Positive psychology, as “the science of positive and constructive subjectivity, strong personality attributes and positive institutions,” increases the quality of life and eliminates disorders that occur when life lacks meaning (32). Constructive organizational behavior emphasizes considering employees' strengths and not their flaws (16). Positive psychological aspects such as optimism and hope and strength and resilience promote the importance of human resources (e.g., a person's expertise and abilities) and social capital (e.g., a person's social networks) in businesses (33). PC is similar to social and human capital in that it is administered and managed, and it can be acquired with relatively modest expenses (20).

Studies of psychological capital (17, 20) define the four aspects of self-efficacy hope, resilience, and optimism. Self-efficacy relates to an individual's perception that a variety of actions can be used to achieve goals (34). An individual's self-efficacy is determined by his or her belief in growth and achievement (35). Self-efficacy alludes to an individual's beliefs regarding their perceived competence to achieve and accomplish goals. Hope is also a major factor that promotes drive, goal attainment, and compatibility (16, 36). Individuals who have high levels of hope are usually confident that they will achieve the things that they desire, so they generate alternate paths to achieve targets if initiative-based strategies are obstructed (31).

Optimism generally entails an emotional and intellectual readiness to accept that positive events are often more significant than negative events (37). An optimistic individual perceives and anticipates the results of everyday experiences in a positive manner (16). Pessimists view similar occurrences as internal, global, and constant, but optimists interpret them as external, variable, and unique. Studies show that resilience is a mechanism or force that boosts tolerance to adversity and anxiety (16, 38). Resilience is a form of growth that allows individuals to keep striving by doing their utmost in the midst of setbacks, adversity, paradoxes, favorable occurrences, advancements, or increased responsibilities (35).

### 2.2. Green innovation ambidexterity

Environmental laws are becoming more strict, so sustainable innovation is equally crucial to preserving sustainability. In addition to positively impacting a company's economic, societal and environmental efficiency, sustainable innovation also addresses environmental problems (11). However, only some businesses have sufficient resources and expertise to promote sustainable innovation (15, 39). Organizations must balance exploitation and exploration to overcome the uncertainty associated with the use of exploration methods and over-reliance on the use of the old practices related to exploitation independently (40).

March (13) was the first to apply the concept of “ambidexterity” to the context of innovation by using exploitation and exploration. Subsequent studies concerned exploratory and exploitative innovation (12, 14, 41–44). If this method improves a company's existing expertise, it is described as exploitative innovation. Exploitative innovation improves existing resources or technological capabilities, and exploratory innovation involves the acquisition of unique and distinct data and insights into integrated systems (14, 41). Exploratory innovation enhances current information and generates new resources or data, so several organizations are investigating data and resources that are external to their organization but which pertain to sustainable innovation (12, 45). Previous studies determined the impact of ambidexterity on a variety of significant organizational issues. These include obtaining the most recent data, enhancing the effectiveness of the organization, developing innovative goods, and management of supply chains (40).

### 2.3. Subjective health

Subjective health (SH) refers to “the many assessments, favorable and unfavorable that individuals make of their lives, and the affective responses of individuals to their personal experience” (16, 46). Therefore, it is usually evaluated using self-reported assessments of a person's emotional situation (affect) or the degree of happiness with various parts of his or her life. Boosting levels of SH throughout society is an important aim of public policy worldwide, and there is an increased emphasis on its assessment and enhancement on a national scale (47). There is also interest in the extent to which improvements to wellbeing and satisfaction benefit the economy (16).

There are valid arguments to demonstrate this. Increasing a person's SH has a meaningful effect on physical wellbeing (48). It boosts creative and problem-solving capabilities and promotes pro-social conduct and higher levels of involvement at work (49). A greater SWB encourages an individual to increase effort or be “smarter.” Recent laboratory studies show a causal connection between better wellbeing and higher efficiency on the individual scale (16, 50).

### 2.4. Psychological health

Employee PH is a complex term that is interpreted in various ways (30). Ryff (51) describes it as a depiction of fitness and health, which is conceptualized as lifelong progressions of development. Schmutte and Ryff (52) describe it as an overarching sense of joy. Panaccio and Vandenberghe (53) define PH as the existence of positive impact, the lack of negative impact, and the occurrence of both work and life satisfaction. Warr (54) operationally defined PH across three aspects: contentment, comfort-anxiety, and enthusiasm-depression.

Diener and Suh (55) noted that PH could involve either ideas or emotions. Hedonic (positive feelings) and eudemonic (positive functionality) wellness are frequently distinguished in discussions of PH (30, 56). Ryan and Deci (26) showed that hedonic wellbeing is often exemplified by life/work contentment and is thought to be motivated by the desire for incentive and or the avoidance of negative events. Eudemonic wellbeing allows self-expression so it is a consequence of the judgment that living circumstances are purposeful, according to Ryff and Keyes's research (57).

Studies show that numerous results are related to PH. Cartwright and Cooper (58) hypothesized that individuals with greater PH levels in the workplace are healthy, friendlier, and live much longer. Wright and Cropanzano (59) noted a positive correlation between PH and work performance. Similar results were presented by Robertson, Birch, and Cooper (60), who noted that PH is more predictive of self-reported levels of efficiency than of positive work and job mindset.

### 2.5. Green performance

Eckersley (61) popularized green theory as a contemporary multidisciplinary method of thought that emphasizes ecology, globalization, ethical responsibility, corporate management, and human rights. Local, national, and global environmental sustainability is the objective of the green theory. Green performance derives its theoretical foundation from green theory and provides crucial data on the integrity of an organization's strategy in terms of environmental requirements (62). It also distinguishes the effectiveness of environmental programs that an organization undertakes.

Yuan and Xiang (63) showed in order to develop into a green corporation; a company must promote environmentally friendly activity by instituting major reforms in product/service standard operating procedures. Manufacturers must abandon the conventional understanding of the product lifecycle from procurement to product creation, distribution, consumption, and disposal (64). Ma et al. (65) showed that businesses that are dedicated to environmentally friendly practices have a significant opportunity to attract additional consumers. Zhang et al. (66) showed that government assistance is necessary to promote firms to transition from old production methods to green sources of business activity.

Li et al. (67) characterized green activity using green technological and sustainable management techniques. Xie et al. (68) categorized green technology as environmentally friendly processes and green innovation. Green methods are administrative efforts to decrease the use of natural resources in manufacturing procedures that convert raw material into something useful (65). It emphasizes the transition from fossil energy to bioenergy or renewable energy (68). By implementing a green approach, businesses guarantee that company operations do not pollute the natural habitat, including the atmosphere, the land, and groundwater (69).

Abbas and Sagsan (70) defined green goods as the advent of unique products/goods or the enhancement of current goods' design features to ensure that the process of production consumes either non-toxic or biodegradable compounds or a minimal amount of non-renewable energy resources in order to maximize energy effectiveness by minimizing discharge or waste. It also relates to product longevity, reusability, the use of eco-friendly raw materials, and the absence of toxic chemicals (71). Green administration relates to the restructuring or incorporation of new management solutions, methods, and regulations that reduce the negative consequences of organizational production and administration techniques and transforms these into eco-friendly processes (67).

### 2.6. Organizational performance

An institution is a team of individuals that have specific duties or positions to accomplish the same purpose by adjusting to and dealing with changing surroundings. Performance refers to the number of objectives that are attained by an institution or an assessment of the efficacy of people, teams, or institutions. On the individual scale, it relates to work satisfaction, accomplished targets, and psychological adjustment. At the collective level, it corresponds to morale, cohesiveness, effectiveness, and performance (72).

Lin (73) noted that productivity is not limited to past accomplishments: it also incorporates the capacity to realize future objectives. Workplace performance includes all behaviors that are connected to organizational goals that vary according to the level of participation of individuals (74). The ultimate objective of an institution is to improve performance to increase corporate effectiveness, which in turn affects an enterprise's potential. Improving organizational performance is the primary objective of every leader in every business. In order to increase organizational performance, it is necessary for a company to develop a complete measurement index that gives employees and the management a clear understanding of the company's current aims and objectives. Currently, the majority of the performance assessment indices use multidimensional performance indicators that are classified as financial or non-financial measurement indices (72). Table 1 lists the meanings and descriptions of all the concepts for this study.

**Table 1:** Meanings of the research concepts.

**Concepts**	**Meanings**	**References**
Psychological capital	PC consists of self-efficacy (SE), hope (HO), optimism (OPT), and resilience (RE), and it enables individuals to be more imaginative and creative in their tasks.	(20)
Green innovation ambidexterity	The concept of “ambidexterity” in the context of green innovation consists of exploitation and exploration. If a method improves the company's existing expertise, it is described as exploitative innovation. Consequently, if the strategy improves enhances current information, and generates new resources or data it is known as explorative.	(39)
Subjective health	The many assessments, favorable and unfavorable, that individuals make of their lives, and also the affective responses of individuals to their personal experience	(46)
Psychological health	PH consists of both hedonic (positive feelings) and eudemonic (positive functionality) wellness. Hedonic wellbeing is often exemplified by life/work contentment and is thought to be motivated by the desire for incentives and or the avoidance of unpleasant events. Eudemonic wellbeing offers the chance to express oneself and therefore is generated from the judgment that one's living circumstances are purposeful.	(51)
Green performance	Green performance derives its theoretical foundation from green theory and gives crucial data on the integrity of an organization's strategy with environmental requirements (62). In addition, it demonstrates the effectiveness and efficacy of environmental programs that an organization has taken.	(75)
Organizational performance	Performance refers to the level of objectives attained by an institution, or an assessment of the efficacy of people, teams, or institutions. On the individual scale, it relates to work satisfaction, accomplished targets, and psychological adjustment. At the collective level, it corresponds to morale, cohesiveness, effectiveness, and performance.	(72)

## 3. Hypothesis development

### 3.1. Psychological capital and green innovation ambidexterity

Individuals who exhibit considerable self-efficacy, ambition, and persistence are capable of proposing and generating new strategies to attain their objectives (16). Bandura (76) showed that SE is the source of the exploitation of original ideas (77). Many studies show a link between self-efficacy in terms of creative management and the progress of companies (16). Hopeful individuals are more inclined to take chances and constantly seek innovative methods to achieve their objectives. These individuals frequently seek creative solutions. In reality, optimistic employees react positively to innovations, irrespective of difficulties at work (20).

Optimists have a more optimistic perspective and anticipate that they will succeed in the future. They generally assume that they affect the events that take place in their lives, so they persist in pursuing original ideas and become more optimistic under pressure. Optimists are also more persistent in their search for solutions and possibilities when they encounter challenges (78). OPT negatively impacts employees' inventiveness and imagination (16, 18). Previous studies show that RE in the office is a reliable indicator of efficiency, attitude toward one's job, and other results in the workplace (16).

Individuals with high RE are more likely to take risks, so they adjust to changing circumstances and highlight and exploit new ideas (20). These individuals mostly seek novel experiences that involve uncertainty and change, so adaptable workers seek new approaches when presented with challenges or setbacks. Risk-taking and creative conduct are modeled for subordinates by adaptive managers (16). Jafri (17) showed that PC and an individual's GIA are highly correlated, so the following hypothesis is postulated:

H1: Psychological Capital has a significant positive effect on Green Innovation Ambidexterity.

### 3.2. Psychological capital and subjective health

SH is an individual's assessment of their own life and has two components: a cognitive component that assesses satisfaction with life and an emotional component that accounts for both positive and negative effects (pleasant and unpleasant emotions and moods) (25, 55). The term “subjective” refers to the participants' inherent viewpoints without imposing any outside frames of reference. It gauges sustained positive emotions rather than transitory moods.

The foundation for the connection is based on the notion that PC includes the personality attributes that affect an individual's SH by providing them with opportunities for personal development, independence, productive work, and positive relationships (79). PC establishes optimism in the workforce and promotes a greater SH. A greater SH results in a variety of positive outcomes, including physical and PH (80), achieving success in significant areas of life and personal aspiration, and managing stress (48).

Employees' PC experiences are imprinted in their emotional intelligence and management and, along with GIA, allow SH to focus on the psychological capabilities and competencies of human capital. The PC of working professionals impacts the SH of operators and the t labor market (25). Cole, Daly, and Mak (79) hypothesized that a person's PC affects employees' SH. It has also been shown that a worker's PC had a positive connection with SH (81). Therefore, the following hypothesis is postulated:

H2: Psychological Capital has a significant positive effect on the Subjective Health of employees.

### 3.3. Psychological capital and psychological health

PH emphasizes skill development and personal development. It is founded on maturity (82), self-actualization (83), and complete functionality (84). The definition of PC highlights the improvement-capable nature of these positive psychological skills. PC has an open structure with growth potential, so it plays a significant part in the development of individuals (85). PC initiates the cognitive, emotional, behavioral, and social processes that lead to PH. It also aids attention, interpretation, and memory processes that are required for domain-specific observations and contentment (86).

Sweetman and Luthans (87) study showed that PC is an individual resource that makes it easier for a person to deal with tough situations and be more proactive, which increases PH and job performance. Ayala and Manzano-García (27) showed that there is a correlation between PH and PC. Luthans et al. (20) noted that PH could be predicted more accurately if PC aspects are viewed as a whole multifunctional entity, as opposed to independent resources. Therefore, the following hypothesis is postulated:

H3: Psychological Capital has a significant positive effect on the Psychological Health of employees.

### 3.4. Green innovation ambidexterity and green performance

The ability of firms to innovate is crucial for survival in the current financial environment (88), but the consequences of corporate transformation are not evenly distributed. There is a very clear distinction between gradual and dramatic innovations. The former pertains to minor changes to existing products, and the latter pertains to brand-new product offers (39). Radical advances are often seen to be the most beneficial in achieving a long-term competitive benefit; incremental advances are also necessary to compete in the short-term market. Businesses must pursue an improvement strategy that emphasizes radical and incremental innovation methods. This is a strategy of “innovation ambidexterity” (42).

Previous studies show that a company can be an omnidirectional organization by seeking both exploitative and experimental inventions (89), and these organizations typically perform better (90). In terms of GIA, GEP or GET is associated with products, processes, and services to protect the environment. It is a process by which businesses continuously launch and implement green initiatives for energy conservation, the prevention of pollution, improving environmental quality, and GP to achieve economic benefits (91). Therefore, GIA is a method by which businesses incorporate environmental issues into their plans and increase their competitive edge by developing technologies that favorably affect their GP (22, 92). Therefore, the following hypothesis is postulated:

H4: Green Innovation Ambidexterity has a significant positive effect on GP.

### 3.5. Subjective health and organizational performance

Individual behaviors, when combined, can have an impact on job performance, so increasing employees' SH can result in financial rewards for businesses. Therefore, the wellbeing of an individual worker can impact work productivity *via* its effect on the worker's productivity and its influence on the output of coworkers. There is a lack of associated employer-employee data to determine the relationship, so there is a paucity of factual studies on the correlation between worker SH and OP. Controlled trials in workplaces or businesses are impractical, and obtaining repeated measurements over time to construct longitudinal datasets is expensive (16).

Studies show a significant association between SH and OP (93). Bartel et al. (94) conducted a longitudinal study of the association between employee behavior and work performance across 193 bank branches in the United States. They showed that branches with happier staff performed better in sales and were less likely to close, but they also noted that these relationships might be explained by other, unnoticed branch characteristics.

In a British insurance company, Proudfoot et al. (95) randomly assigned 81 individuals from a sample of 136 individuals to a training program to raise employees' levels of self-esteem and work satisfaction and reduce their level of mental discomfort. Three months after the intervention, a follow-up study showed that SH was greater in the intervention group than in the control group. The intervention group also had lower employee turnover, and 2 years later, their OP had increased (16). Therefore, the following hypothesis is postulated:

H5: Subjective Health has a significant positive effect on Organizational Performance.

**Figure 1:**
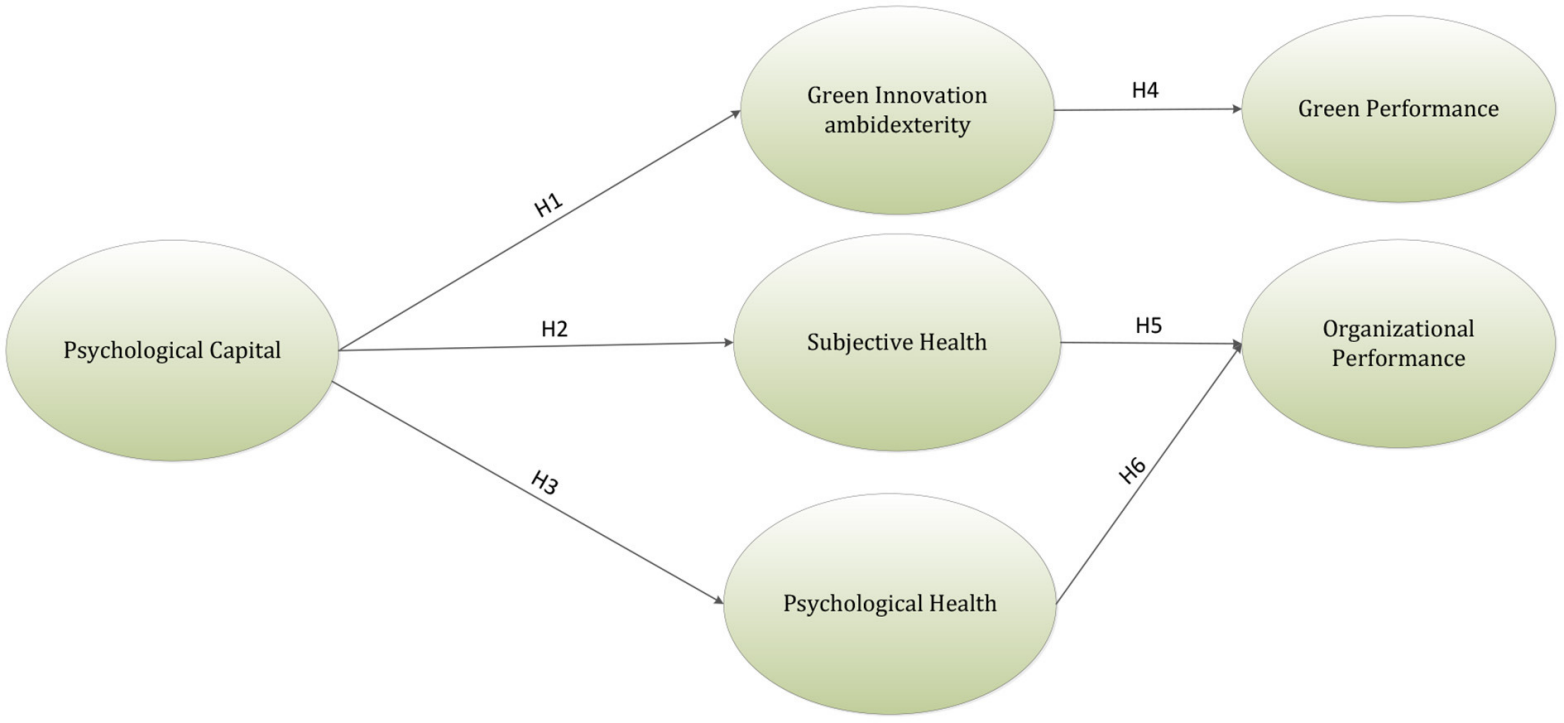
Theoretical framework.

Figure 1 indicates the theoretical framework of the research.

### 3.6. Psychological health and organizational performance

Studies show that employee PH and OP have a significant effect, particularly if they support employees' professional and personal development (96). Most learning techniques aim to maximize workers' capacities to boost job participation and OP, but these techniques can also allow workers to perform better by giving them the tools to better deal with stress by developing emotional intelligence (97).

Mierlo et al. (98) showed that learning-promoting behaviors counteract the negative effects of work intensification. Learning techniques that energize individuals through task design reduce emotional tiredness and boost PH (99). Other “functional” human resource (HR) practices involve expanding and improving employee engagement and voice practices to comprehend employees' PH needs and to communicate OP requirements that ultimately allow the discovery of mutual gain thresholds, so compensation and benefits packages can be tailored to provide equitable pay scales and staff with spiritual vitality and ethical character are hired (100). PH Schemes such as Occupational safety and health programs, including counseling to enhance PH, reduce absenteeism, and increase OP (30, 101).

Recent studies show that in order for businesses to increase their OP, they must consider employee PH after changes in working conditions, including the introduction of information technology, financial insecurity, and economic, political, and global upheavals. Therefore, employers establish procedures that promote employee PH (30, 56). The following hypothesis is postulated:

H6: Psychological Health has a significant positive effect on Organizational Performance.

## 4. Research methodology

The information for this study is collected from a variety of sources in the Chinese GB industry. The subjects for this study are Chinese employees of GB-based companies. Using a convenience sampling method, a total of 504 employees were selected. The employees' answers to a restricted, self-administered survey were used to gather the data. A total of 90.41% of the subjects responded, which is a strong response rate (102). Likert scale inquiries elicited the degree of agreement rather than agreement or disagreement with a statement. This study uses a 7-point Likert scale, with 7 denoting strong agreement, 1 denoting strong disagreement, and 4 denoting neutrality.

There are issues with the reliability of the points that are included in a Likert scale. Previous studies show that a 5-to-7-point Likert scale is a reliable measurement tool, and a 10-point Likert scale is less reliable because it provides a lower score than a 5 or 7-point Likert scale (39).

The objects from the study by Youssef, Luthans and Avolio (78) were modified and used to measure the PC. Items from Darvishmotevali et al.'s (103) study were adjusted to measure SH. PH is measured by adapting Klar et al.'s (104) research. GIA was studied using objects from Khan et al.'s (42) research. GP was studied using the items from Úbeda-García et al.'s (22) research. OP was studied using items modified from Lee and Tseng's (72) research. A pilot test using 100 randomly selected representative sample employees was conducted. The questionnaire forms were assessed and validated using the results of this test.

## 5. Data analysis

The PLS method (partial least squares) was used to evaluate the data. This study has two parts. The validity and reliability of the constructs were evaluated in the study's preliminary stage, and the second stage determined the constructs' causal orientations and the path coefficients (105). PLS is the best technique for maintaining the proposed connections and for computing complicated research frameworks (106). The availability of effective indicators to deal with randomization in a study's results allows PLS to be used to quantify outcomes that feature an unconventional distribution. Dynamic methods were examined (107, 108). PLS is preferable to other SEM methodologies for data analysis for this study.

### 5.1. Convergent validity

PLS-SEM is used for examining complicated models (109). Table 1 demonstrates that the standardized factor loadings of each indicator range from 0.71 to 0.98, with all values exceeding the minimum threshold value of 0.5, so each indicator has high reliability and validity (110). Rho_A, Cronbach's Alpha, CR, and AVE, i.e., are used to measure the Convergent Validity of the study. Cronbach's alpha is used to verify internal consistency, and rho_A and CR are indices for reliability. Instead of comparing loadings, Rho_A determines the instrument's reliability by comparing the weights (111). Cronbach's Alpha and Rho_A must be more than 0.7 to be regarded as credible (112).

Table 2 demonstrates that, as anticipated, all constructs have Cronbach's Alpha and Rho_A values larger than 0.7. All components also have CR values that are >0.7 (113), so the instrument has internal validity, and the results correlate with the outcomes in Table 2. The average variance for each construct, which is a factor of the AVE, is used to calculate the composite reliability. This construct only has substantial convergent validity if the value is >0.5 (114). The hypothetical construct variables' AVEs range from 0.742 to 0.908 (as shown in Table 2), which demonstrates a high level of convergence.

**Table 2:** Convergent validity of the outer model.

**Constructs**	**Indicators**	**Factor loading**	**Cronbach's alpha**	**rho_A**	**Composite reliability**	**Average variance extracted (AVE)**
SE	SE1	0.832	0.892	0.892	0.920	0.698
	SE2	0.837				
	SE3	0.861				
	SE4	0.845				
	SE5	0.802				
OPT	OPT1	0.800	0.840	0.864	0.889	0.621
	OPT2	0.839				
	OPT3	0.861				
	OPT4	0.860				
HO	HO1	0.837	0.923	0.925	0.942	0.766
	HO2	0.897				
	HO3	0.904				
	HO4	0.876				
	HO5	0.861				
RE	RE1	0.890	0.946	0.947	0.959	0.823
	RE2	0.901				
	RE3	0.895				
	RE4	0.940				
	RE5	0.908				
GET	GET1	0.947	0.975	0.975	0.980	0.909
	GET2	0.953				
	GET3	0.943				
	GET4	0.962				
	GET5	0.961				
GEP	GEP1	0.955	0.976	0.976	0.982	0.933
	GEP2	0.965				
	GEP3	0.971				
	GEP4	0.973				
FP	FP1	0.981	0.981	0.981	0.988	0.963
	FP2	0.980				
	FP3	0.983				
NFP	NFP1	0.962	0.994	0.994	0.994	0.940
	NFP2	0.968				
	NFP3	0.962				
	NFP4	0.967				
	NFP5	0.969				
	NFP6	0.975				
	NFP7	0.971				
	NFP8	0.974				
	NFP9	0.972				
	NFP10	0.972				
GP	GP1	0.967	0.980	0.980	0.985	0.944
	GP2	0.970				
	GP3	0.980				
	GP4	0.969				
PH	PH1	0.710	0.926	0.938	0.945	0.778
	PH2	0.913				
	PH3	0.913				
	PH4	0.937				
	PH5	0.915				
SH	SH1	0.896	0.922	0.924	0.945	0.811
	SH2	0.925				
	SH3	0.888				
	SH4	0.892				

FP, Financial performance; NFP, Non-Financial performance; SE, Self-efficacy; HO, Hope; OPT, Optimism; RE, Resilience; GEP, Green Exploratory Innovation; GET, Green Exploitative Innovation; GP, Green performance; PH, Psychological health; SH, Subjective health.

### 5.2. Discriminant validity

This refers to how distinct two constructs are from one another. The study's discriminant validity is calculated using the Fornell and Larcker criteria. The square root of the average variance extracted, or AVE, is used to determine latent variables (115). Table 3 demonstrates that the components are more effective than other indicators in describing the variation because the square root of adjusted variance estimates (AVEs) are greater than those for competing constructs.

**Table 3:** Fornell-Larcker criterion.

**Constructs**	**FP**	**GIA**	**GET**	**GEP**	**GP**	**HO**	**NFP**	**OPT**	**OP**	**PC**	**PH**	**RE**	**SE**	**SH**
FP	0.920													
GIA	0.381	0.720												
GET	0.327	0.862	0.858											
GEP	0.306	0.798	0.382	0.871										
GP	0.522	0.618	0.451	0.589	0.857									
HO	0.219	0.496	0.473	0.342	0.374	0.845								
NFP	0.530	0.623	0.529	0.506	0.621	0.368	0.797							
OPT	0.258	0.346	0.293	0.282	0.414	0.451	0.489	0.749						
OP	0.706	0.621	0.528	0.503	0.656	0.365	0.975	0.476	0.751					
PC	0.275	0.555	0.476	0.445	0.517	0.748	0.555	0.797	0.536	0.643				
PH	0.302	0.544	0.450	0.457	0.399	0.554	0.428	0.493	0.436	0.614	0.739			
RE	0.198	0.408	0.351	0.325	0.385	0.426	0.479	0.551	0.452	0.799	0.396	0.861		
SE	0.196	0.481	0.366	0.440	0.455	0.461	0.416	0.571	0.399	0.806	0.494	0.508	0.813	
SH	0.462	0.311	0.269	0.248	0.449	0.312	0.569	0.488	0.597	0.475	0.397	0.354	0.361	0.813

FP, Financial performance; NFP, Non-Financial performance; SE, Self-efficacy; HO, Hope; OPT, Optimism; RE, Resilience; GEP, Green Exploratory Innovation; GET, Green Exploitative Innovation; GP, Green performance; PH, Psychological health; SH, Subjective health.

The discriminant validity determines the distinction between the various constructs and study items. Table 4 shows that the indicators that are used to assess the constructs have adequate discriminant validity. Every indicator measuring a single construct has a factor loading value that is greater than that for all other constructs in the latent structure. In Table 4, the highest value of each indicator is highlighted in yellow (116).

**Table 4:** Cross loadings.

**Constructs**	**PC**	**SH**	**PH**	**GIA**	**GP**	**OP**
**SE1**	0.668	0.276	0.301	0.237	0.202	0.149
SE2	0.711	0.341	0.351	0.294	0.249	0.204
SE3	0.723	0.343	0.380	0.305	0.255	0.219
SE4	0.727	0.344	0.380	0.327	0.298	0.242
SE5	0.728	0.369	0.436	0.392	0.338	0.293
OPT1	0.701	0.450	0.471	0.388	0.368	0.352
OPT2	0.762	0.470	0.490	0.455	0.391	0.388
OPT3	0.790	0.535	0.549	0.475	0.417	0.415
OPT4	0.787	0.567	0.544	0.520	0.460	0.466
OPT5	0.491	0.492	0.419	0.438	0.431	0.414
HO1	0.806	0.614	0.633	0.593	0.567	0.554
HO2	0.864	0.597	0.686	0.670	0.617	0.585
HO3	0.872	0.638	0.673	0.650	0.605	0.592
HO4	0.838	0.608	0.708	0.689	0.645	0.617
HO5	0.810	0.650	0.691	0.686	0.646	0.628
RE1	0.838	0.726	0.736	0.732	0.682	0.687
RE2	0.866	0.662	0.757	0.753	0.717	0.703
RE3	0.806	0.728	0.735	0.736	0.707	0.704
RE4	0.891	0.716	0.790	0.786	0.745	0.743
RE5	0.819	0.707	0.778	0.757	0.720	0.741
SH1	0.662	0.896	0.753	0.699	0.683	0.717
SH2	0.704	0.925	0.809	0.752	0.742	0.768
SH3	0.594	0.888	0.742	0.702	0.683	0.737
SH4	0.597	0.892	0.728	0.688	0.677	0.719
PH1	0.420	0.664	0.710	0.672	0.686	0.717
PH2	0.738	0.728	0.913	0.828	0.801	0.816
PH3	0.694	0.778	0.913	0.827	0.815	0.827
PH4	0.752	0.794	0.937	0.854	0.836	0.845
PH5	0.697	0.750	0.915	0.821	0.784	0.829
GET1	0.684	0.739	0.872	0.938	0.868	0.883
GET2	0.722	0.743	0.861	0.939	0.876	0.878
GET3	0.692	0.768	0.842	0.926	0.862	0.885
GET4	0.709	0.745	0.865	0.954	0.902	0.907
GET5	0.684	0.776	0.876	0.955	0.925	0.916
GEP1	0.631	0.715	0.855	0.939	0.917	0.893
GEP2	0.668	0.747	0.848	0.946	0.893	0.897
GEP3	0.693	0.737	0.867	0.956	0.914	0.913
GEP4	0.663	0.752	0.866	0.957	0.926	0.916
GP1	0.692	0.776	0.880	0.937	0.967	0.923
GP2	0.626	0.745	0.839	0.911	0.970	0.905
GP3	0.667	0.737	0.877	0.931	0.980	0.917
GP4	0.614	0.748	0.865	0.912	0.969	0.913
FP1	0.569	0.797	0.876	0.883	0.894	0.936
FP2	0.557	0.780	0.856	0.876	0.883	0.936
FP3	0.575	0.783	0.870	0.878	0.890	0.948
NFP1	0.628	0.808	0.878	0.922	0.896	0.967
NFP2	0.624	0.794	0.873	0.914	0.885	0.960
NFP3	0.629	0.776	0.876	0.917	0.906	0.964
NFP4	0.609	0.785	0.875	0.914	0.912	0.965
NFP5	0.682	0.798	0.896	0.928	0.917	0.969
NFP6	0.645	0.783	0.888	0.919	0.909	0.964
NFP7	0.666	0.783	0.895	0.932	0.921	0.969
NFP8	0.624	0.770	0.875	0.920	0.908	0.971
NFP9	0.640	0.779	0.894	0.926	0.911	0.969
NFP10	0.625	0.772	0.879	0.922	0.914	0.968

FP, Financial performance; NFP, Non-Financial performance; SE, Self-efficacy; HO, Hope; OPT, Optimism; RE, Resilience; GEP, Green Exploratory Innovation; GET, Green Exploitative Innovation; GP, Green performance; PH, Psychological health; SH, Subjective health. The highest value of each indicator is highlighted in yellow.

### 5.3. Empirical results

Using Smart PLS 3.2.8, a path analysis of the research framework assessment was conducted. During this phase, the inner model was computed. A *t*-value and a *p*-value are used to determine the assumptions for the internal model. The hypotheses are supported if the *t*-value exceeds 1.96 and the *p*-value is < 0.05. R square represents the ratio of predictor constructs, which is then used to demonstrate the study framework's predictive power (40, 117, 118). Therefore, the R square value is significant if the value is ~0.67, moderate if the value is ~0.33, and weak if the value is ~0.19 (113). To further ensure a framework's predictive accuracy, Q square values are determined and analyzed. The Q square values for this study were obtained using the blindfolding technique in SMART PLS. The predictive accuracy of a construct is validated if the value of the Q square is >0 (119).

**Figure 2:**
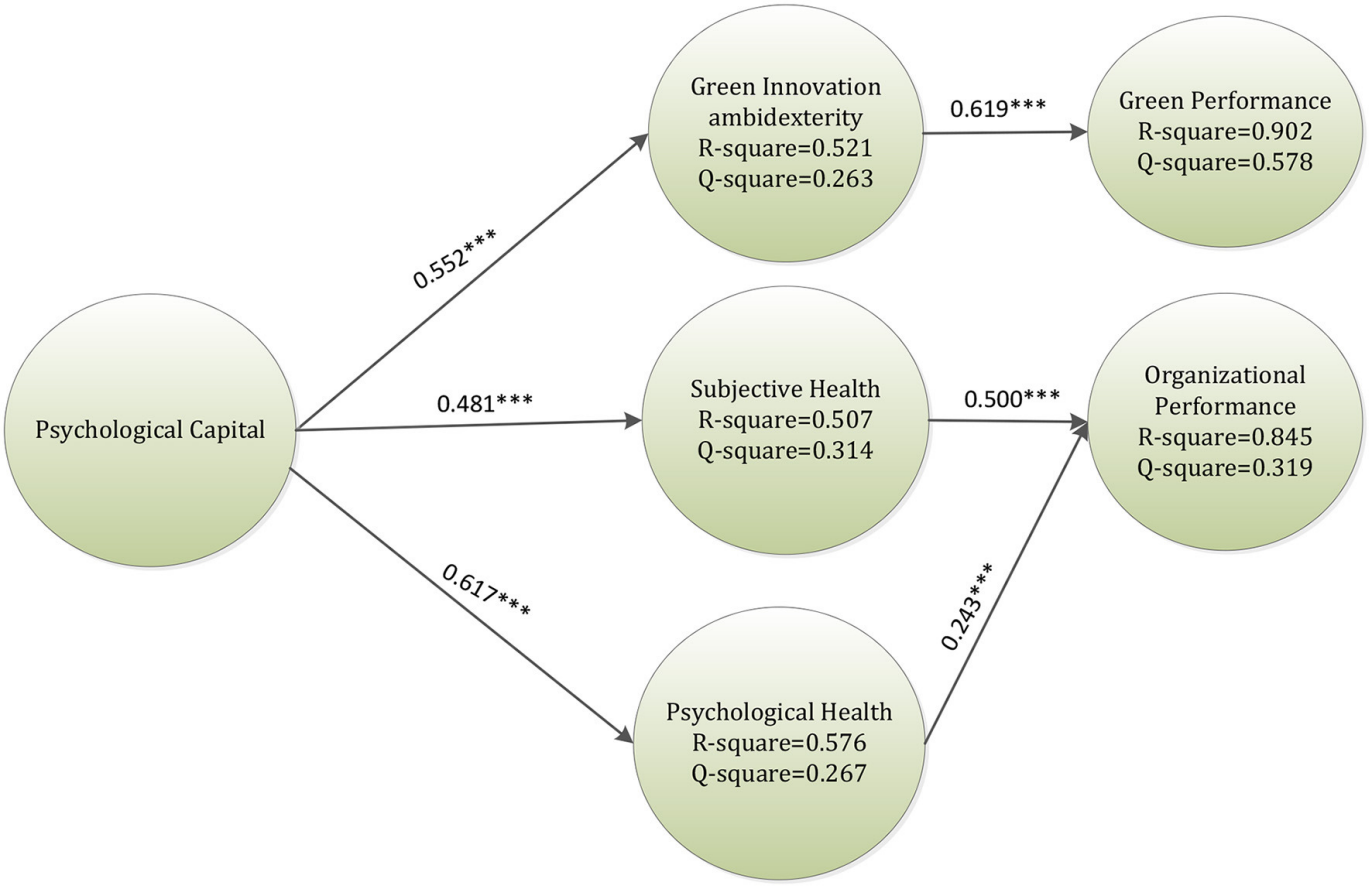
Research results. ***p < 0.001.

The empirical results are shown in Table 5 and Figure 2. Figure 2 indicates the *p*-values (^***^p < 0.001) of the relationships. Data from this study shows that PC has a significant effect on GIA (β = 0.552, *t*-value = 10.263), SH (β = 0.481, *t*-value = 10.005), and PH (β = 0.617, *t*-value = 9.512) so H1, H2, and H3 are supported. GIA has a significant effect on GP, so H4 is also supported (β = 0.619, *t*-value = 13.156). Furthermore, SH has a significant impact on OP, so H5 is supported (β = 0.500, *t*-value = 8.099). PH has a significant association with OP, so H6 is supported (β = 0.243, *t*-value = 3.825).

**Table 5:** Empirical results.

**Hypotheses**	**Path coefficient (β)**	***T*-values**	**P-values**
H1: PC -> GIA	0.552	10.263	0.000
H2: PC -> SH	0.481	10.005	0.000
H3: PC -> PH	0.617	9.512	0.000
H4: GIA -> GP	0.619	13.156	0.000
H5: SH -> OP	0.500	8.099	0.000
H6: PH -> OP	0.243	3.825	0.000

PC, Psychological Capital; GIA, Green Innovation Ambidexterity; GP, Green performance; PH, Psychological health; SH, Subjective health.

This study investigated the indirect effects by analyzing the specific indirect effects provided by Smartpls. According to the results, the *p*-values (^**^p < 0.01; ^***^p < 0.001) of all the proposed indirect relationships were less than 0.05, which indicated that the indirect relationships were statistically significant. Furthermore, this study also employed the Sobel test to generate the *Z* values and assess the indirect paths' statistical significance (120). The findings of the Sobel test provided significant *p*-values, and the values were found to be >1.96, which further indicated the significance of the proposed indirect relationships, as shown in Table 6 of the study. Moreover, the study also utilized the Bootstrapping approach and generated 95% confidence intervals of the specified indirect relationships. Consequently, none of the confidence intervals included 0. Hence, the significance of the indirect relationships was further confirmed (121, 122).

**Table 6:** Indirect effects.

**Indirect effects**	**Direct path**	***T*-value of direct path**	**S. D**.	***z*-value of Sobel test**	**Indirect path coefficient**	***T*-value of indirect path**	**Bias-corrected percentile bootstrap confidence interval (95%)**
PC -> SH -> OP	PC -> SH	10.005	0.047	6.294^***^	0.239^***^	6.017	(0.555, 0.679)
	SH -> OP	8.099	0.063				
PC -> PH -> OP	PC -> PH	9.512	0.059	3.548^***^	0.145^**^	2.829	(0.712, 0.572)
	PH -> OP	3.825	0.063				
PC -> GIA -> GP	PC -> GIA	10.263	0.052	8.092^***^	0.343^***^	6.563	(0.659, 0.696)
	GIA -> GP	13.156	0.043				

PC, Psychological Capital; GIA, Green Innovation Ambidexterity; GP, Green performance; PH, Psychological health; SH, Subjective health.

^**^p < 0.01;

^***^p < 0.001.

## 6. Discussion and conclusions

The theoretical framework for this study is developed using PC, self-determination theory, and green theory. This study addresses research gaps. The effect of PC on PH, GIA, and SH is analyzed. Furthermore, the effect of GIA on GP is determined. Finally, the effect of PH and SH on OP is also determined.

The results of this study show that PC significantly affects GIA. The results are in agreement with those of an earlier study by Ziyae et al. (16), which determined the effect of PC on IT advancement for Agriculture Bank branches in Tehran, Iran, for 132 managers and staff members who worked in Tehran's Agriculture Bank branches. The data analysis method used was SEM. The study's findings show that PC has a significant effect on IT innovation. Further study showed that, with the exception of RE, none of the psychological capital dimensions, including HO, SE, RE, and OPT, enhance IT innovation. This study determines a method to increase PC and staff capabilities through ongoing measurement and the use of improvement initiatives in order to foster greater IT innovation.

The results of this study also show that PC and SH have a significant relationship. The results of this study are in good agreement with those of the study by Rastogi and Singhal (25), which determined PC's function as a determinant of SH and work motivation. The results of a quantifiable survey-based research design that used information from 300 Indian employees showed that PC is a determinant for SWB and CC. This study showed that supervisors must train workers who have a higher incidence of PC to increase their SH if they are to produce a workforce that is dedicated to careers and, by extension, professions. Workers who achieved states of SE, OPT, RE, and HO are also strongly inclined to have higher levels of life satisfaction, positive effects, and a lower degree of negative effects, so they make the necessary commitment to their careers and maintain their employment.

The results of this study also show that there is a strong connection between PH and PC. The results are in agreement with those of Ayala and Manzano-García's (27) earlier studies, which determined how burnout functions as a mediator between PH and PC in the direct support workers in specialized autistic services. SEM was used to test the model based on the hypothesis. The results show that PC has a significant effect on PH and that PC at work reduces burnout, which increases PH. The results show that in order to minimize burnout and attain a higher PWB, initiatives that enhance each person's PC must be put into place.

The results of this study also show that GIA has a substantial effect on GP. The results are in agreement with those of Úbeda-García et al.'s (22) study. Recent research in the field of environmental management shows staff behavior has a significant effect on GP, but few studies determine the connection between HR and GP (22). The study by Úbeda-García et al. (22) determined the association between GPWS and GP through mediating GIA. A sample of Spanish hotel companies was subjected to SEM-PLS, and the results show that GHPWS encourages the growth of GIA and that this parameter increases GP.

The results of this study show that SH does not significantly affect OP. The results of this study are different from those of the study by Bryson et al. (28), which determined the connection between workers' SH and OP. These studies demonstrated a distinct, favorable, and statistically significant association between SH and OP. This study also demonstrates an association between OP and PH, which is in agreement with the results of the study by Loon et al. (30), which determined how employees' PH and OP are related. This study demonstrated the inconsistencies in employee OP and PH practices, a way to address the tension that exists between OP and PH by creating a fresh conceptual framework, and it distinguished between endogenous factors that organizations can influence to improve the synergies between employee PH and OP practices, providing a more nuanced perspective to the dominant arguments.

The results of this study show that the indirect relationships of PC → PH → OP and PC → SH → OP are significant. The results can be compared to an earlier study by Rastogi and Singhal (25), which determined the role of SH as a mediator between career commitment and PC and showed that SH partially mediates the connection between career commitment and PC. The study showed that SH is an important mediator.

Finally, according to the findings of the study, the indirect relationship of PC → GIA → GP is also found to be significant. The results are somewhat comparable with those of the study by Seman et al. (123). The growing environmental consciousness of the public and the application of government legislation compel businesses to incorporate organizational environmentally-friendly practices, such as GSCM and GIA (123). Seman et al.'s (123) study provided empirical proof that GSCM and GIA significantly enhance GP to motivate organizations to adopt these practices. PLS-SEM analysis shows that there is a favorable and statistically significant association between GP, GSCM, and GIA. GIA also has a beneficial effect on GP and is a mediator between GSCM and GP.

## 7. Theoretical implications

The results of this study give information about GIA in GB industries, and the PC dimensions OPT, HO, RE, and SE, individually. In order to increase SH, PH, and OP, staff members' capacities and PC must be increased through ongoing measurement and the use of improvement programs (16). Therefore, this study uses green theory, self-determination theory, and PC to develop a theoretical framework. This study measures the effect of PC on GIA, SH, and PH, it measures the effect of GIA on GP, and it determines the effect of SH and PH on OP.

This study also contributes to the notion of positive psychology by uniquely studying PC with SH and PH in the context of GB industries. In the green construction sector, the analysis of PC within a business is a relatively new discipline. Salanova (124) noted that in order to achieve optimal OP, it is increasingly clear that companies must capitalize on employees' abilities. For positive organizational psychology, a worker's PH and SH depict genuine goals that must be reflected in organizational policies. In order to increase OP and allow employees to feel psychologically fulfilled, individual strengths and psychological competencies must be measured, developed, and managed.

This study adds to the sparse body of research on the relationship between GIA and an organization's GP. Previous studies show that GIA affects GP (125). This study demonstrates that GIA's dynamic capability enhances GP. In this sense, the study adds to the body of knowledge by affirming that GIA enhances GP and that cultivating and promoting green capabilities is crucial for manufacturing industries and the green construction industry. Therefore, service companies must emphasize the integration, construction, and reconfiguration of external and internal resources that ultimately lead to increases in GEP and GET for products, services, or processes that are related to protecting the environment, in order to cultivate the dynamic capacity of GIA (22).

## 8. Practical implications

The green building industry sector must also support fresh concepts in order to gain a competitive edge by implementing and promoting innovative cultures, especially in terms of service delivery and customer understanding, because innovation is critical for these industries. The components of the intra-correlations between the PC dimensions can be improved by fostering and enforcing a productive work environment in their workplace to improve PH, SH, and GIA. The sum of all PC dimensions means that policymakers must focus on all components rather than specific elements. The green building industry must adopt a systematic strategy to improve the PC of personnel (16).

This study also demonstrates that GIA is essential for GP in businesses, and in order to improve GP, green processes, products, and service innovation must be innovative and must decrease or eliminate harmful environmental impacts (22). The results of this study show how to increase employees' PH. Previous empirical studies showed that the PC is a developmentally open condition and that short training micro-interventions increase it (126). Training programs improve employees' sentiments in terms of PH and SH and job satisfaction, which reduces staff turnover and sick days (27).

Avey et al. (127) noted that a small investment in PC significantly improves the PH of employees. To improve PC, this investment might include workshops, individual and group counseling, and support groups. It is necessary to establish problem-solving techniques that enable staff members to improve and build their PC and reduce burnout. Training sessions should be planned throughout the year, with timetables dependent on the workers' availability (27).

The results of this study show that in order to ensure their own health, those who manage the green construction industry must improve the PH of their personnel. A health office that supports employees and provides individualized monitoring of weaknesses and strengths is of particular use in this respect.

## 9. Limitations and future research

The results of this research must be evaluated from the perspective of its constraints. Future research could evaluate the OP and GP consequences of GIA innovation in the green building sector and the variables such as SH and PH that affect the indirect associations between PC and GP, and OP, as demonstrated by this study. Future studies might also be comparative in nature to determine aspects that influence innovations in the GB sector (16). Future studies should also seek a deeper comprehension of the link between PC and GP, and OP.

This study uses data for a specific point in time. Future research might use a longitudinal design to determine this association more accurately. The details of the connections between the contextual factors and specific green HR practices that are needed to make GIA must be derived. To determine mechanisms for GIA and GP, a more nuanced evaluation of the various types of green participation programs, green selection programs, and green reward programs is required (22).

This study uses a small sample, but it is sufficient to test the hypotheses. It pertains to the Chinese green design industry sector. The model could be used for other fields if larger samples from a wider range of industries were used. This study pertains to GB industries in China, and the results are limited to a specific industry. Future studies might involve major and trending industries in China.

This study analyzed the proposed indirect impacts but fell short of analyzing the mediating roles of PH, SH, and GIA due to the lack of empirical evidence to support the direct relationships of PC with OP and GP. Hence, future researchers are encouraged to propose and analyze the aforementioned direct relationships once there is enough supportive empirical evidence. Furthermore, once they have analyzed the direct relationships, potential researchers are suggested to find the mediating roles of PH, SH, and GIA.

This study pertains to China which is an emerging economy. Future studies might use the same model for other emerging economies or test the model in a developed economy. Future studies might also compare emerging and developing economies by comparing the results of this study with the results of a developed economy.

## Data availability statement

The raw data supporting the conclusions of this article will be made available by the authors, without undue reservation.

## Ethics statement

Ethical review and approval was not required for the study on human participants in accordance with the local legislation and institutional requirements. Written informed consent from the participants was not required to participate in this study in accordance with the national legislation and the institutional requirements.

## Author contributions

Conceptualization, formal analysis, investigation, writing—original draft, and writing—review and editing: S-XC and YT. Both authors have read and agreed to the published version of the manuscript.
